# *Vitex simplicifolia* Mitigates Cadmium‐Induced Alzheimer‐like Neurotoxicity through Enzymatic and Neurotransmitter Modulation in Animal Model

**DOI:** 10.1002/alz70861_108671

**Published:** 2025-12-23

**Authors:** Ifeoma Felicia Chukwuma, Victor Onukwube Apeh, Timothy Prince Chidike Ezeorba

**Affiliations:** ^1^ University of Nigeria, Nsukka, Nsukka, Enugu Nigeria; ^2^ Federal University of Allied Health Sciences, Enugu, Enugu Nigeria; ^3^ University of Birmingham, Edgbaston, Birmingham UK

## Abstract

**Background:**

Cadmium (Cd) disrupt neurotransmitter homeostasis, leading to significant neurotoxicity. This study investigated the neuroprotective effects of *Vitex simplicifolia* extract in a CdCl₂‐induced neurotoxicity model.

**Method:**

Twenty‐five male Wistar rats were divided into five groups. Group 1 (normal control), while groups 2–5 were administered CdCl₂ (5 mg/kg body weight). Group 2 (untreated), groups 3–5 received ascorbic acid (20 mg/kg b.w.) or *V. simplicifolia* extract at 200 and 400 mg/kg b.w., respectively, for 21 days via oral administration. Biochemical and histological analyses of brain tissues were performed to assess neurotransmitter levels, enzymatic biomarkers, and neuronal integrity.

**Result:**

CdCl₂ exposure significantly (p < 0.05) decreased levels of dopamine (2.88 ± 0.64 ng/100 mL), serotonin (21.45 ± 1.83 ng/mL), acetylcholine (229.34 ± 12.10 U/L), glutamate dehydrogenase (1.03 ± 0.041 U/L), and Na⁺/K⁺‐ATPase activity (2.32 ± 0.52 nmol Pi/min), while increasing monoamine oxidase activity (0.03 ± 0.01 mg/mL) and GABA levels (8.32 ± 0.63 mg/mL), in CdCl₂ model compared to the control group values of 6.37 ± 0.53 ng/100 mL, 42.66 ± 2.98 ng/mL, 299.05 ± 17.18 U/L, 1.91 ± 0.08 U/L, 4.47 ± 0.72 nmol Pi/min, 0.04 ± 0.04 mg/mL, and 5.49 ± 0.48 mg/mL, respectively. Treatment with the extract significantly reversed these neurochemical alterations, restoring neurotransmitter and enzyme levels toward normal. Histological assessments revealed that the extract ameliorated neuronal damage by reducing necrotic cell bodies seen in CdCl₂ exposured rats

**Conclusion:**

Thus, *V. simplicifolia* may serve as a promising therapeutic candidate for mitigating Cd‐induced neurotoxicity through modulation of neurotransmitter levels and enzymatic biomarkers.

